# Statistical Analysis of Hurst Exponents of Essential/Nonessential Genes in 33 Bacterial Genomes

**DOI:** 10.1371/journal.pone.0129716

**Published:** 2015-06-12

**Authors:** Xiao Liu, Baojin Wang, Luo Xu

**Affiliations:** College of Communication Engineering, Chongqing University, 174 ShaPingBa District, Chongqing, 400044, China; University of Helsinki, FINLAND

## Abstract

Methods for identifying essential genes currently depend predominantly on biochemical experiments. However, there is demand for improved computational methods for determining gene essentiality. In this study, we used the Hurst exponent, a characteristic parameter to describe long-range correlation in DNA, and analyzed its distribution in 33 bacterial genomes. In most genomes (31 out of 33) the significance levels of the Hurst exponents of the essential genes were significantly higher than for the corresponding full-gene-set, whereas the significance levels of the Hurst exponents of the nonessential genes remained unchanged or increased only slightly. All of the Hurst exponents of essential genes followed a normal distribution, with one exception. We therefore propose that the distribution feature of Hurst exponents of essential genes can be used as a classification index for essential gene prediction in bacteria. For computer-aided design in the field of synthetic biology, this feature can build a restraint for pre- or post-design checking of bacterial essential genes. Moreover, considering the relationship between gene essentiality and evolution, the Hurst exponents could be used as a descriptive parameter related to evolutionary level, or be added to the annotation of each gene.

## Introduction

Essential genes are those genes of an organism that are critical for its survival, and their content are highly determined by the circumstances in which the organism lives [1]. Essential genes are characterized by lethal mutant phenotypes that block survival or reproduction. Identifying essential genes is important for understanding the minimal requirements for cellular survival, and this information may have practical applications in the fields of medicine and bioengineering [2]. Furthermore, this information may provide insight into the complex mechanisms of cell function and evolution.

The identification of essential genes still relies predominantly on biochemical experiments. Single-gene-specific mutagenesis, saturation transposon mutagenesis, and antisense RNA inhibition are employed as traditional approaches [3]. However, these methods are costly, time-consuming, and labor-intensive. In addition, experimental approaches are not always possible as the vast majority of organisms are unculturable. Computational prediction methods therefore offer a good alternative. Many methods for essential gene prediction have previously been reported; these include comparative genomic approaches using homology mapping, constraint-based methods, machine learning methods based on a partial essential gene list, and statistical modeling based on transposon mutagenesis data [4]. To increase prediction accuracy, researchers employed a large range of features, such as amino acid composition, codon bias, and protein evolution rate [2,4]. However, the accuracy of these methods is not always high enough for large scale practical application. Moreover, the increase in the number of features (the dimension of feature space) results in a sharp increase in computational complexity and computational cost [5], which highlights the necessity for more efficient methods and feature(s).

Long-range correlation (or self-similarity) has been observed in DNA sequences, in both coding and non-coding regions [6,7]. The Hurst exponent is used as a characteristic parameter to describe long-range correlation. Yu *et al*. proposed a time-series model and a visual representation for DNA sequence analysis [8]. The correlation dimension, Hurst exponent, fractal dimension, and dimension spectrum (multifractal analysis) were considered in their work. Liu *et al*. calculated the rescaled range functions; the Hurst exponents of human chromosome 22 and enterobacteria phage lambda DNA sequences; and the transmission coefficients, Landauer resistances, and Lyapunov coefficients of segments of the corresponding DNA sequences [9]. Stan *et al*. investigated the characteristics of a series of lengths of coding and non-coding DNA sequences from certain bacteria and archaea, using the generalized Hurst exponent on the size of fluctuations, the shape of the singularity spectra, the shape and relative disposition of the curves of the singular measures scaling exponent, and the values of the associated parameters [10].

With the rapid development of synthetic biology, especially research on minimal genomes, the question has arisen as to whether these long-range correlation features change in reduced DNA sequences. We performed an initial study of the Hurst exponent of nine bacteria from the DEG 6.5 database (the original database of essential genes) and obtained an initial view of the distribution feature of the index, that is, a normal distribution exists for the Hurst exponent [11]. However, the sample size was limited in that study. The updated DEG 10.7 database, released more recently, contains 33 bacteria, allowing for a more comprehensive study [12]. Here, we investigated the distribution of the Hurst exponent of 33 bacteria from the DEG 10.7 database to analyze the long-range correlation of this feature.

## Materials and Methods

The essential gene lists of 33 bacterial objects were downloaded from the DEG 10.7 database, and their genome files and sequence data were obtained from the NCBI ftp site (ftp://ftp.ncbi.nih.gov/genomes/Bacteria/). Sequence information is listed in Table 1.

**Table 1:** Information of the analyzed objects.

Analysis organisms	NCBI RefSeq access number	Gene number (Full-gene-set)	Gene number (Essential)	Gene number (Nonessential)
*Acinetobacter baylyi* ADP1	NC_005966	3307	499	2594
*Bacillus subtilis* 168	NC_000964	4175	271	3904
*Bacteroides fragilis* 638R	NC_016776	4290	547	3743
*Bacteroides thetaiotaomicron* VPI-5482	NC_004663	4778	325	4453
*Burkholderia pseudomallei* K96243	NC_006350/006351	3398+2329	505	5222
*Burkholderia thailandensis* E264	NC_007650/007651	3276+2356	406	5226
*Campylobacter jejuni subsp*. *jejuni* NCTC 11168 = ATCC 700819	NC_002163	1576	228	1395
*Caulobacter crescentus*	NC_011916	3885	480	3224
*Escherichia coli* MG1655 I	NC_000913	4140	609	2923
*Escherichia coli* MG1655 II	NC_000913	4140	296	4077
*Francisella novicida* U112	NC_008601	1719	392	1329
*Haemophilus influenzae* Rd KW20	NC_000907	1610	642	512
*Helicobacter pylori* 26695	NC_000915	1469	323	1135
*Mycobacterium tuberculosis* H37Rv	NC_000962	3906	614	2552
*Mycobacterium tuberculosis* H37Rv II	NC_000962	3906	771	3171
*Mycobacterium tuberculosis* H37Rv III	NC_000962	3906	687	3070
*Mycoplasma genitalium* G37	NC_000908	475	381	94
*Mycoplasma pulmonis* UAB CTIP	NC_002771	782	310	322
*Porphyromonas gingivalis* ATCC 33277	NC_010729	2089	463	1627
*Pseudomonas aeruginosa* PAO1	NC_002516	5572	117	5454
*Pseudomonas aeruginosa* UCBPP-PA14	NC_008463	5892	335	960
*Salmonella enterica serovar Typhi*	NC_004631	4352	353	4005
*Salmonella enterica serovar Typhi* Ty2	NC_004631	4352	358	3906
*Salmonella enterica serovar Typhimurium* SL1344	NC_016810	4446	353	4035
*Salmonella enterica subsp*. *enterica serovar Typhimurium str*. 14028S	NC_016856	5315	105	5210
*Salmonella typhimurium* LT2	NC_003197	4451	230	4228
*Shewanella oneidensis* MR-1	NC_004347	4065	403	1103
*Sphingomonas wittichii* RW1	NC_009511	4850	535	4315
*Staphylococcus aureus* N315	NC_002745	2582	302	2281
*Staphylococcus aureus* NCTC 8325	NC_007795	2767	351	2541
*Streptococcus pneumoniae*	NC_003098	1813	244	NULL^a^
*Streptococcus sanguinis*	NC_009009	2270	218	2052
*Vibrio cholerae* N16961	NC_002505/002506	2534+970	779	2943

^a^ NO nonessential genes information provided in DEG.

It should be noted that there are actually 33 bacterial object sequences provided in the DEG database, with six object sequences originating from three bacteria (*E*. *coli* MG1655, *Mycobacterium tuberculosis* H37Rv, and *S*. *enterica serovar Typhi*) according to different experimental environments and methods (See S1 and S2 Datasets or http://tubic.tju.edu.cn/deg/organism.php?db=p). The organism number is therefore actually 29. Moreover, three objects have two chromosomal sequences (*Burkholderia pseudomallei* K96243, *Burkholderia thailandensis* E264, and *V*. *cholerae* N16961).

### Analytical procedures

The data were analyzed according to the following steps:

1. Sequences were digitized.

Nucleotide sequences were transformed into digital sequences by expressing each nucleotide as a digital number. The four nucleotides, A, G, C, and T, were assigned the digital numbers 0, 1, 2, and 3, respectively (e.g., DNA sequence AGCTTTT would be digitized as 0123333).

1. Hurst exponents were analyzed using the R software.

The Hurst exponent describes the degree of self-similarity of a data set. The Hurst exponent of a data series with long-range dependence is between 0.5 and 1. An increased Hurst exponent indicates an increase in the degree of self-similarity and long-range dependence [13].

The Hurst exponents of each gene (including the full-gene-set, essential genes, and nonessential genes) were calculated using the R software [14]. To compare the feature in various models, six approaches (nine modes) for Hurst exponents were employed, which are [15]:

- RoverS, which estimates the Hurst exponent using the rescaled range (R/S) method.
- hurstSpec in standard, smoothed, and robinson modes, which estimates the Hurst exponent via spectral regression.
- hurstACVF, which estimates the Hurst exponent by regression of scaled asinh plot of autocovariance function vs. log(lag).
- Detrended fluctuation analysis, which estimates the scaling exponent from the results.
- FDWhittle, which analyzes the input time series with the spectral density function.
- Block in aggvar and higuchi mode, which estimates the Hurst exponent in the time domain.

1. Statistical analysis with SPSS.

The distribution properties of the Hurst exponents of the full-gene-set, the essential genes, and the nonessential genes of the 33 object sequences were analyzed using SPSS (IBM, Armonk, NY). Two methods in SPSS were used. Q–Q plots present an intuitionistic and graphic result. However, Q–Q plots lack a quantitative description of the data. Therefore, significance levels based on the Kolmogorov–Smirnov (K–S) test, were also calculated, which evaluated whether the datasets were significantly different from an assumed theoretical distribution.

## Results and Discussion

A distribution hypothesis is accepted when it has a significance level of greater than or equal to 0.05, and is rejected when the significance level is less than 0.05. Of the four hypothetical distributions, that is, normal, uniform, Poisson, and exponential distribution, our results showed that only a normal distribution was satisfied at various levels. The hurstSpec method in smoothed mode provided the highest significance level among the nine methods, and was chosen as our optimal analysis method (See S1 and S2 Datasets in supplementary materials for detail). The results are listed in Table 2.

**Table 2:** Significance levels of 33 objects in a normal distribution based on the hurstSpec method in smoothed mode.

Analysis organisms	NCBI RefSeq access number	Full-gene-set	Essential Genes	Nonessential Genes
*Acinetobacter baylyi* ADP1	NC_005966	0.052	0.604	0.093
*Bacillus subtilis* 168	NC_000964	0.004	0.439	0.004
*Bacteroides fragilis* 638R	NC_016776	0.002	0.175	0.015
*Bacteroides thetaiotaomicron* VPI-5482	NC_004663	0.000	0.688	0.000
*Burkholderia pseudomallei* K96243	NC_006350/006351	0.000	0.645	0.001
*Burkholderia thailandensis* E264	NC_007650/007651	0.000	0.408	0.000
*Campylobacter jejuni subsp*. *jejuni* NCTC 11168 = ATCC 700819	NC_002163	0.018	0.192	0.074
*Caulobacter crescentus*	NC_011916	0.000	0.757	0.000
*Escherichia coli* MG1655 I	NC_000913	0.000	0.807	0.075
*Escherichia coli* MG1655 II	NC_000913	0.000	0.639	0.000
*Francisella novicida* U112	NC_008601	0.045	0.258	0.089
*Haemophilus influenzae* Rd KW20	NC_000907	0.037	0.711	0.291
*Helicobacter pylori* 26695	NC_000915	0.289	0.324	0.394
*Mycobacterium tuberculosis* H37Rv	NC_000962	0.000	0.717	0.009
*Mycobacterium tuberculosis* H37Rv II	NC_000962	0.000	0.431	0.001
*Mycobacterium tuberculosis* H37Rv III	NC_000962	0.000	0.845	0.004
*Mycoplasma genitalium* G37	NC_000908	0.996	0.993	0.662
*Mycoplasma pulmonis* UAB CTIP	NC_002771	0.131	0.894	0.133
*Porphyromonas gingivalis* ATCC 33277	NC_010729	0.000	0.343	0.000
*Pseudomonas aeruginosa* PAO1	NC_002516	0.001	0.978	0.001
*Pseudomonas aeruginosa* UCBPP-PA14	NC_008463	0.001	0.289	0.181
*Salmonella enterica serovar Typhi*	NC_004631	0.001	0.183	0.002
*Salmonella enterica serovar Typhi* Ty2	NC_004631	0.001	0.503	0.024
*Salmonella enterica serovar Typhimurium* SL1344	NC_016810	0.006	0.421	0.015
*Salmonella enterica subsp*. *enterica serovar Typhimurium str*. 14028S	NC_016856	0.000	0.904	0.000
*Salmonella typhimurium* LT2	NC_003197	0.003	0.516	0.004
*Shewanella oneidensis* MR-1	NC_004347	0.014	0.784	0.212
*Sphingomonas wittichii* RW1	NC_009511	0.002	0.167	0.005
*Staphylococcus aureus* N315	NC_002745	0.004	0.437	0.013
*Staphylococcus aureus* NCTC 8325	NC_007795	0.000	0.124	0.002
*Streptococcus pneumoniae*	NC_003098	0.220	0.177	NULL
*Streptococcus sanguinis*	NC_009009	0.009	0.127	0.020
*Vibrio cholerae* N16961	NC_002505/002506	0.002	0.000	0.053

The significance levels of the Hurst exponents for essential genes were significantly greater than those of the corresponding full-gene-set, except for *Mycoplasma genitalium* G37, *Streptococcus pneumoniae*, and *Vibrio cholerae* N16961. For example, the significance levels of *Escherichia coli* MG1655 I were <0.001 and 0.807, respectively. Those of *Salmonella enterica subsp*. *enterica serovar Typhimurium str*. 14028S were <0.001 and 0.904, respectively. These results indicate that a normal distribution exists for the Hurst exponent of the essential genes in these organisms. Even for the two exceptions, the significance levels of *M*. *genitalium* G37 (0.996, 0.993) and *S*. *pneumoniae* (0.220, 0.177) were significantly greater than 0.005, which means that a normal distribution also exists for the Hurst exponent of these essential genes.

*V*. *cholerae* N16961 was different, showing a significance level of <0.001 for the Hurst exponents of essential genes. In fact, *V*. *cholerae* strain C6706 was used for analysis and the results were compared with *V*. *cholerae* strain N16961 [16]. Although only 50–250 single nucleotide polymorphisms were detected across the two entire genomes, the cause of these differences remains to be determined [17].

From our results, the significance levels of most of the nonessential genes remained unchanged or increased slightly compared with those of the corresponding full gene set. For example, the significance levels of *E*. *coli* MG1655 I increased slightly (0.075), whereas those of *S*. *enterica subsp*. *enterica serovar Typhimurium str*. 14028S remained unchanged (<0.001). There was one exception, *Helicobacter pylori* 26695, for which the significance levels of nonessential genes were greater than those of both the full gene set and the essential genes. However, the three values were all significantly greater than 0.005, indicating that the Hurst exponents of the essential genes still followed a normal distribution.

It should be addressed that the genomes of *Mycoplasma genitalium* G37 and *Mycoplasma pulmonis* UAB CTIP are already near minimal. Although the other 31 genomes encoded much more genomic redundancy than *Mycoplasma genitalium* G37 and *Mycoplasma pulmonis* UAB CTIP, the Hurst exponents followed the same distribution (normal distribution), which showed the generality of the Hurst exponents.

**Fig 1:**
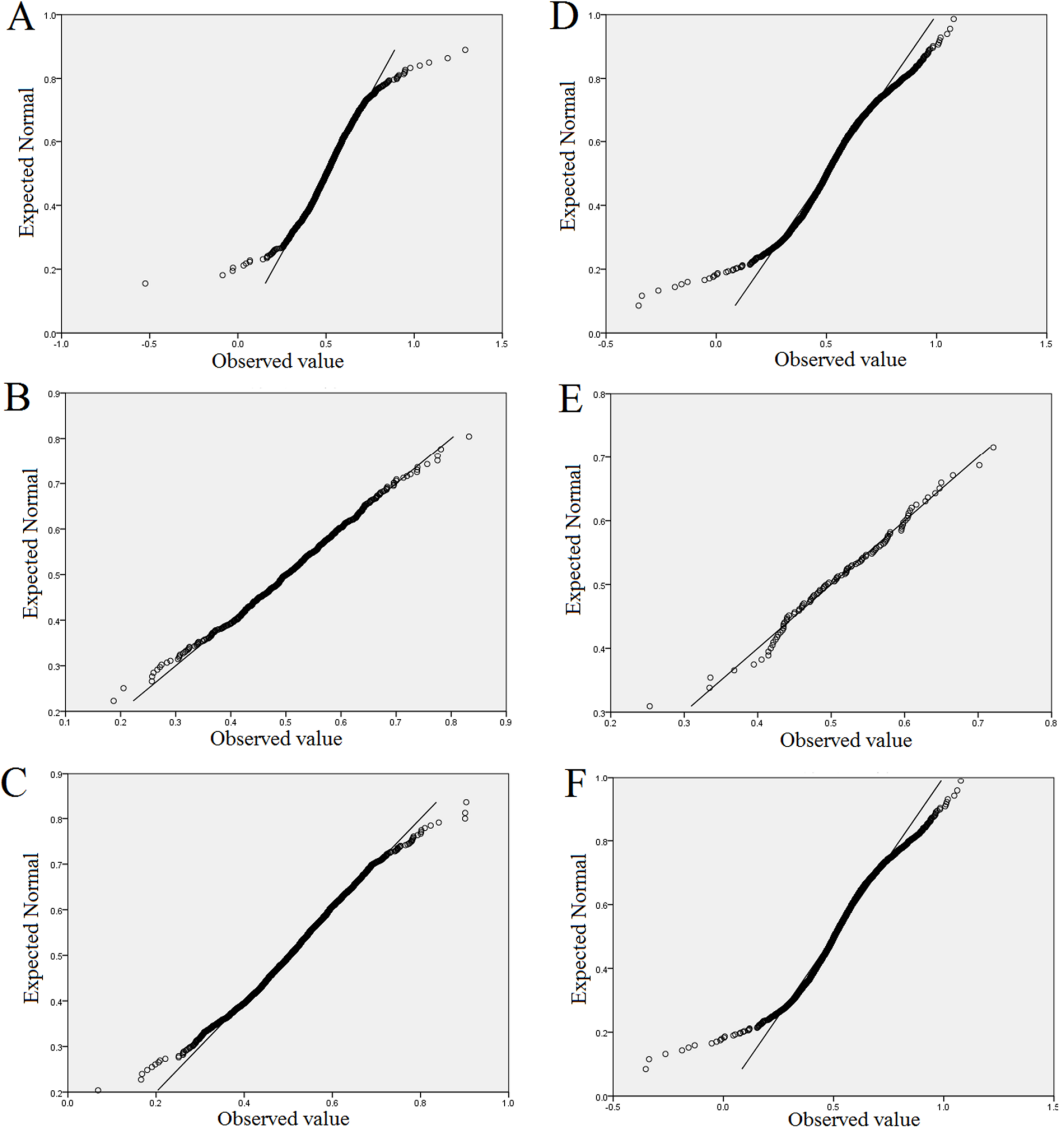
Q–Q plots of the Hurst exponents. A, B, and C: *Escherichia coli* MG1655 I; D, E, and F: *Salmonella enterica subsp*. *enterica serovar Typhimurium str*. 14028S. A and D show Q–Q plots of the Hurst exponents of the full-gene-sets of the two objects from respective organisms. B and E show Q–Q plots of the Hurst exponents of the essential genes of the two objects from respective organisms. C and F show Q–Q plots of the Hurst exponents of the nonessential genes of the two objects from respective organisms.

Q–Q (quantile–quantile) plots were also generated from our data set. In a Q–Q plot, if the data investigated follow a normal distribution, a straight line should be produced when they are plotted against the expected data. Because of the lack of quantitative descriptions, we only provide the results of two objects in Fig 1, *E*. *coli* MG1655 I and *S*. *enterica subsp*. *enterica serovar Typhimurium str*. 14028S. The essential gene data clearly departed less from linearity than the corresponding full gene set, indicating that the essential gene data followed a normal distribution more closely. However, the nonessential genes of most objects were relatively conserved with the full-gene-set.

## Conclusion

There are more than 5000 bacterial genomes currently available in the NCBI sequence database, far more than the number of objects provided in the DEG database. There is a demand for improved computational prediction methods for determining gene essentiality. The aim of our study was to identify the shared feature(s) related to gene essentiality and to use these to aid *in silico* predictions. We investigated the statistical feature of Hurst exponents in the 33 available genomes. The results showed that in most organisms (31 out of 33) the significance levels of the Hurst exponents of the essential genes were significantly higher than for the corresponding full-gene-set. In most organisms, the significance levels of the Hurst exponents of the nonessential genes remained unchanged or increased only slightly. With only one exception, the Hurst exponents of essential genes followed a normal distribution.

Machine learning methods have previously been employed for predicting gene essentiality. Various sequence-based and biochemistry-based characteristics have been chosen as descriptive features, including GC content, codon effective number, topology features of protein-protein interactions, gene expression, cellular localization, and biological process annotation [4]. To increase prediction accuracy, more efficient features should be explored. The final goal of computational prediction methods is to reduce or even eliminate the dependency on biochemical experiments. Thus, some of the features used currently should be avoided, such as protein-protein interactions and gene expression, which cannot be derived from the sequence directly. Based on our findings, the distribution feature of Hurst exponents show its generality in the essential genes of bacteria, therefore we propose that the distribution feature of Hurst exponents of essential genes could be used as an effective index for data classifying or clustering in the prediction of essential genes of bacteria. The Hurst exponent has been screened out and adopted as key feature in our related support vector machine-based prediction method and the results verified the efficiency of the Hurst exponent (unpublished results).

As essential genes code for fundamental cellular functions required for the viability of an organism, they tend to be more conserved than nonessential genes across organisms, which means lower evolutionary rates [18,19]. Furthermore, it has been reported that long-range correlations increase with evolution [20]. This means that the Hurst exponents of essential/nonessential genes are a descriptive parameter related to evolutionary level, which could be added to the annotation of each gene. Quantitative analysis of the relationship between the Hurst exponents of essential genes and the evolution of essential genes will be the subject of our future work.

Under different environmental conditions, a gene may gain or lose essentiality. This has prompted research into conditionally essential genes, which will expand our knowledge of which genes in an organism are essential and under what conditions they are essential [21,22]. Readers can find the detail of the laboratory condition in DEG (http://tubic.tju.edu.cn/deg/organism.php?db=p) or S1 and S2 Datasets. It’s a pity that, at present, there are not enough contrasting data of the objects in variant laboratory conditions for classifying or clustering, highlighting the need for a database of conditionally essential genes.

Experimental confirmation of data derived from computational prediction methods is still indispensable for the accurate prediction of essential genes. However, the development and improvement of specific databases, such as DEG, will help to increase the prediction accuracy of computational approaches.

## Supporting Information

S1 Dataset Hurst exponents of the essential genes of the 33 bacteria in DEG10.7. (XLSX)

S2 Dataset Hurst exponents of the nonessential genes of the 33 bacteria in DEG10.7. (XLSX)
